# Focus and trends in nurse advocacy in the Pan American Health Region: a bibliometric analysis*

**DOI:** 10.1590/1518-8345.4368.3312

**Published:** 2020-08-31

**Authors:** David Charles Benton, Alyson Suzanne Brenton

**Affiliations:** 1 National Council of State Boards of Nursing, Chicago, Illinois, United States of America.; 2 George Washington University, School of Nursing, Washington, District of Columbia, United States of America.

**Keywords:** Bibliometrics, Pan American Health Organization, Health Policy, Health Advocacy, Scholarly Communication, Social Networking, Bibliometria, Organização Pan-Americana da Saúde, Política de Saúde, Advocacia em Saúde, Comunicação Acadêmica, Rede Social, Bibliometría, Organización Panamericana de la Salud, Política de Salud, Defensa de la Salud, Comunicación Académica, Red Social

## Abstract

**Objective:**

this study examined scholarly output relating to nursing advocacy contributions toward influencing policy by authors in countries of the Pan American Health Organization.

**Method:**

the study utilizes a bibliographic analysis of papers indexed in Scopus authored by PAHO member state scholars. VOSviewer conducted coauthor and cooccurrence analysis to generate visualizations of the relationships between authors, countries of origin and keywords.

**Results:**

7,773 papers with 21,523 authors met the inclusion criteria. An increase of publications on policy starting in 1962 was found. Co-authorship identified a fragile relationships structure with few authors bridging networks of collaboration. By country of origin, 22 of 35 member states contributed to policy literature; 17 in a connected network and 5 contributing but neither connected to peers nor other member states. Keyword analysis identified 20 specific data clusters.

**Conclusion:**

our findings are aligned with the Nursing Now Campaign. This bibliographic analysis provides an important benchmark into current policy advocacy activity in PAHO against which future progress in the region can be assessed. There is scope for greater collaboration amongst authors and this could be targeted toward engagement of nurses in member states not-yet or only partially active in this space.

## Abstract (pt)

**Objetivo:**

examinar os resultados acadêmicos relacionados às contribuições do ativismo de enfermagem para influenciar políticas de autores em países da Organização Pan-Americana da Saúde.

**Método:**

o estudo utiliza uma análise bibliográfica de artigos indexados no Scopus de autoria de estudiosos dos Estados-membros da OPAS. O VOSviewer conduziu análises de coautor e de co-ocorrência para gerar visualizações das relações entre autores, países de origem e palavras-chave.

**Resultados:**

7.773 artigos com 21.523 autores preencheram os critérios de inclusão. Foi encontrado um aumento de publicações sobre políticas a partir de 1962. Na coautoria, identificou-se uma estrutura de relacionamento frágil com poucos autores conectados em redes de colaboração. Por país de origem, 22 dos 35 Estados-membros contribuíram para a literatura sobre políticas; 17 estavam em uma rede conectada e cinco contribuíam, mas não estavam conectados a colegas nem a outros Estados-membros. A análise de palavras-chave identificou 20 agrupamentos de dados específicos.

**Conclusão:**

nossas descobertas estão alinhadas com a Campanha *Nursing Now*(Enfermagem Agora) e essa análise bibliográfica fornece uma referência importante para a atual atividade de ativismo político na OPAS, em relação à qual o progresso futuro na região pode ser avaliado. Observa-se a possibilidade de maior colaboração entre autores e isso pode ser direcionado ao envolvimento de enfermeiros nos Estados-membros que ainda não participam ou participam apenas parcialmente nesse espaço.

## Abstract (es)

**Objetivo:**

este estudio examinó producciones académicas relacionadas con aportes a la defensa de la Enfermería con vistas a influenciar las políticas a cargo de autores que viven en países de la Organización Panamericana de Salud.

**Método:**

en el estudio se emplea un análisis bibliográfico de trabajos indexados en Scopus con autoría de académicos de los Estados Miembro de la OPS. En VOSviewer se realizó un análisis de co-autoría y co-aparición para generar visualizaciones de las relaciones entre autores, países de origen y palabras clave.

**Resultados:**

7.773 trabajos de 21.523 autores cumplieron con los criterios de inclusión. Se descubrió un aumento en la cantidad de publicaciones sobre políticas desde 1962. En cuanto a la co-autoría se identificó una estructura de relaciones frágil en la cual pocos autores participaban en redes de colaboración. Por país de origen, 22 de 35 Estados Miembro aportaron a la literatura sobre políticas; 17 en una red conectada y 5 con aportes pero no conectados a colegas ni a otros Estados Miembro. En el análisis de palabras clave se identificaron 20 agrupamientos de datos específicos.

**Conclusión:**

nuestros hallazgos se condicen con la Campaña *Nursing Now* (La Enfermería hoy); este análisis bibliográfico proporciona un importante punto de referencia sobre la actividad actual de defensa de las políticas en la OPS con respecto al cual se podrá evaluar el progreso a futuro en la región. Se dispone de buen campo para una mayor colaboración entre autores y esto podría dirigirse a la participación activa de las enfermeras de los países miembro que todavía no participan activamente en este espacio, o que solo lo hacen en forma parcial.

## Introduction

2020, sees the celebration of 200 years since the birth of Florence Nightingale^(^ 1 ^)^. Over the two centuries since her birth, the profession of nursing has evolved considerably not least as a result of the innovative actions of Nightingale herself^(^ 2 ^)^. Hence, we will argue that this is a time to reflect and acknowledge on both our successes and failures but importantly look to the future to describe what the profession can achieve in the years ahead.

It has been reported that Lord Nigel Crisp has suggested that the actions of the World Health Organization (WHO) naming 2020 as the *International Year of the Nurse and Midwife* provides a once in a generation opportunity for governments to really show nurses and midwives how much they are valued^(^ 3 ^)^. To do this, the profession needs to play its part. In today’s era of evidence-based policy there is a need to curate our existing contributions, acknowledge their impact and promote a vision of what is yet to come. The Nursing Now Campaign has initiated a social movement encouraging the profession to bring its expertise and voice to the policy table^(^ 4 ^)^. To be an effective voice, nurses must come prepared to offer evidence, suggest solutions and embrace the opportunity to shop-window our contributions throughout and beyond the many celebrations stimulated by the WHO Year of the Nurse and Midwife designation.

In recent years the Pan American Health Organization (PAHO), along with other intergovernmental and national governmental bodies, have increased their reliance on the use of evidence in their policy making processes^(^ 5 ^-^ 6 ^)^. PAHO has also been active in producing a wide range of policy documents targeted at priority health challenges such as non-communicable disease as well as more widely addressing the role and contribution of professions like nursing^(^ 7 ^-^ 8 ^)^.

To address the challenges and objectives^(^ 7 ^)^ set in the PAHO strategies on human resources for universal access to health there is a need for the profession to answer the call of the Nursing Now Campaign. Historically, nurses have advocated for those we care for as well as for the advancement of profession through practice, research and policy change. Ever since Florence Nightingale, advocacy has been foundational to the success of the profession. Indeed, for nursing to continue to advance we must build on past successes to shape and improve health care as we speed towards the attainment of the sustainable development goals^(^ 9 ^)^. To do this we must understand the contribution of nurses in the Pan American Health Region have made up until this point. By doing this we can identify the foundation we have already established as well as identifying the opportunities for future success. Mapping past scholarship on how nurses influence and advocate for change will provide the knowledge needed to drive forward efforts to shape current and future evidence-based education, practice, regulation and policy.

## Method

This mixed method bibliographic study examines the published and indexed output of scholarship relating to the profession of nursing written by authors based in the countries of the Pan American Health Organization. The intent is to provide a high-level analysis of the origins and thematic content of the work so as to identify current strengths as well as future opportunities for further scholarship.

While bibliometric analysis has been used extensively in the information sciences it is only recently that nurse scholars have used the method on a regular basis^(^ 10 ^)^. One of the early protagonists for the technique of bibliometrics^(^ 11 ^)^ described the approach as one that applies statistical methods and mathematics to the collation of the content of books, articles and other communication. Accordingly, it provides a means of synthesizing the content of published work to determine general themes, the evolution of thinking and metrics based on the most prolific authors, frequency of citation and published sources^(^ 12 ^)^. Additionally it has been argued, that visualization of data that incorporates the use of proximity (closely related values are located near to each other), color (signifying related items being grouped into a single cluster), size of nodes (offering an indication of frequency), and thickness of links (providing a marker of strength of relationships), can present images that engage the viewer and make information more comprehensible^(^ 13 ^)^.

To create bibliometric visualizations data for such studies requires comprehensive information on the articles published as well as any citations that the papers have accrued. This can be obtained from three major sources, Scopus (Elsevier), Web of Science (Clarivate Analytics) or Google Scholar. While both Scopus and Web of Science are curated databases (there is a fixed list of sources that are systematically indexed) Google Scholar uses a web crawling technique and hence the coverage of sources is unknown and therefore was rejected. Of the two remaining option Scopus has the most comprehensive coverage of nursing content and as a result was the database of choice so as to optimize the likelihood of PAHO nurse scholarship discovery.

To extract data from Scopus a document search of the entire database using standard keyword, wildcard and delimiters was used. The search took place on January 28, 2020 and used the search string TITLE-ABS-KEY ((“Advocac*” OR “Influenc*”) AND (“policy” OR “Politic*” OR “Legislat*” OR “Guidance” OR “Guide*” OR “Model*” OR “Framework”) AND LIMIT-TO (SUBJARE, “NURS”)). Subsequent to this search the resulting list of articles was further limited by selection of those papers that were identified as being authored by a person or persons from a PAHO member state.

The identified papers were then downloaded as a comma separated value (CSV) file ready for import to the analytical software. In this case, VOSviewer, a freeware package developed by the University of Leiden, generates a visualization of relationships between the variables of interest. Multivariate scaling techniques were used to calculate relationships between authors, their countries of origin and the keyword themes used by the authors to describe their work^(^ 14 ^)^. A frequency threshold of three occurrences of the name/theme was set as this has been suggested as the minimum frequency to identify meaningful clusters^(^ 15 ^)^. In addition, Excel 365 was used as a means of graphing and calculating the general trend associated with the frequency of publications indexed and retrieved from Scopus.

## Results

**Figure 1:**
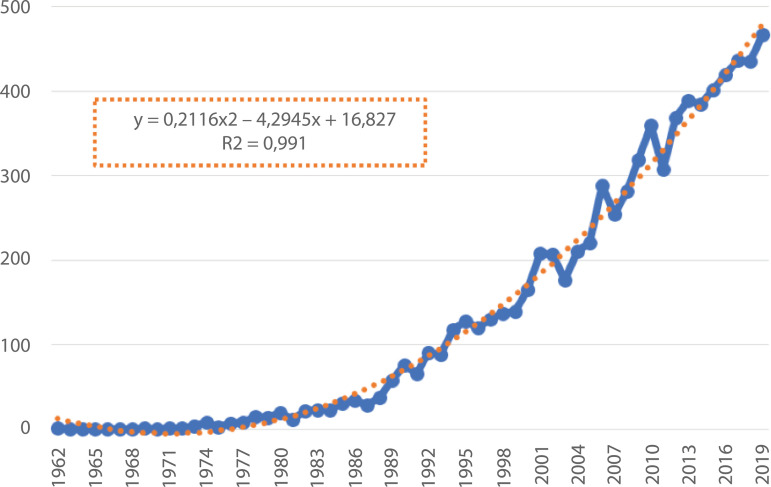
Frequency of papers produced and indexed in Scopus

A total of 7,773 papers met the inclusion criteria. The first paper, identified by the search strategy, focused on policy advocacy, and published by an author from a PAHO member state dates to 1962. The 7,773 papers were authored by a total of 21,523 individuals. Figure 1 illustrates the frequency of production of papers by authors from a PAHO country or territory. By inspection there has been a significant increase over the years and by superimposing a trend line the output of papers from 1962 to 2019 can be accurately described by use of the polynomial equation, $y=0.2116x^{2}\;-\;4.2945x\;+\;16.827$ with an R^2^ value of 0.991.

**Figure 2:**
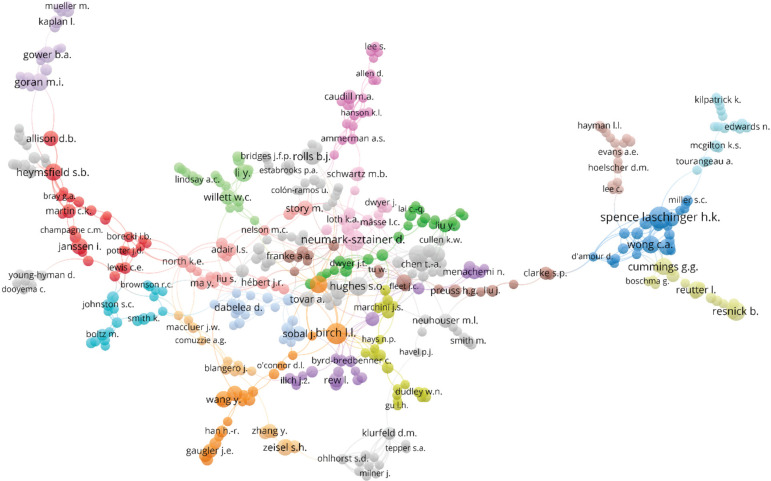
Co-authorship network of authors who have published 3 or more papers

To identify connections between authors a co-authorship analysis was conducted and revealed, of those authors that had contributed to three or more papers (932 in total), 440 of them had worked together with one or more peers at some point from Jan 1, 1962 up until Jan 28 2020 (the date that data was extracted from the Scopus database). However, the network of authors is quite tenuous as indicated by the presence of a few authors that bridge two or more sub-networks of collaboration. If any of these individuals were lost, such as Clarke SP, (see node, one quarter of distance from right hand side of the image) then the network would fragment into two or more sub-networks. In short, individuals such as Clarke SP, broker connections between two or more groups of scholars (Figure 2).

**Figure 3:**
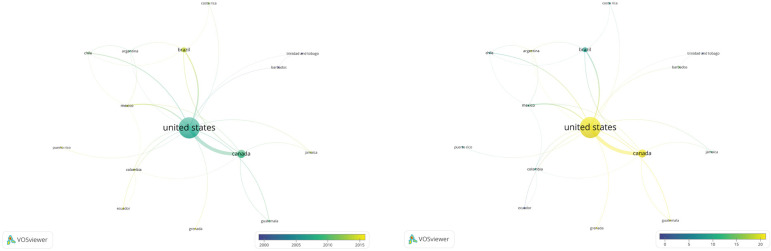
Average year of publication (left) & average citation rate by country (right)

Further examination of the authorship network, this time analyzed by country of author origin, identifies that of the 35 Member States of the PAHO region, 22 have contributed to the nursing-based policy advocacy literature (Figure 3).

Of the 22 Member States where authors live or work, 17 of these along with three other nations/territories (United Kingdom, South Korea and Puerto Rico) are part of a connected network (Figure 3). The five PAHO countries that have contributed to the literature but are not connected, either to each other through collaborative scholarship or to the network through collaboration on publications, are: Belize, Cuba, Dominica, Paraguay and Peru.

Furthermore, Figure 3 illustrates that the United States and Canada are the most prolific contributors. This is perhaps unsurprising since graduate and post-graduate programs of study and research have been established in these countries for many decades. However, looking at the left-hand image, it is positive to note that several countries are relatively new contributors to the literature such as Dominican Republic, Ecuador, and Grenada and hence provides concrete evidence of scholarly progress.

Examination of the right-hand image, documenting the average rate of citation for papers originating in the various countries illustrates that the most cited papers on this topic come from the United States, Canada and Grenada.

**Figure 4:**
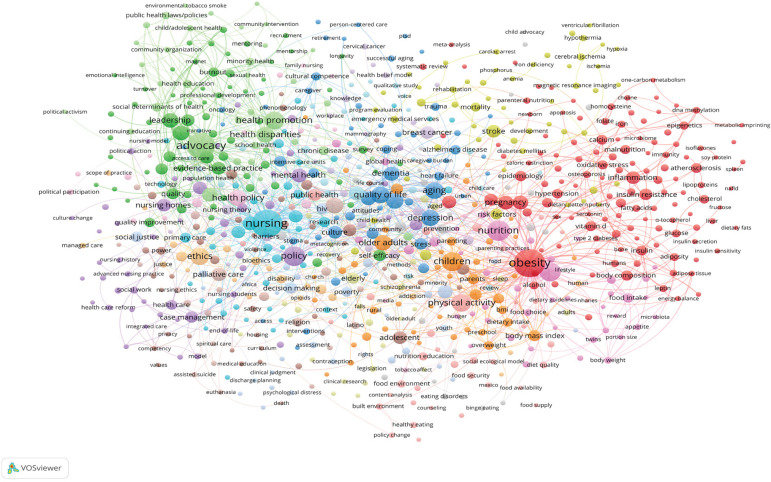
Co-occurrence analysis of author key words

Figure 4 details a co-occurrence cluster analysis of author defined keywords. Related work is identified through use of common keywords specified by the authors to summarize the foci of their papers. A total of 11,702 keywords were provided by the authors of which 813 met the frequency threshold (three or more occurrences). The 813 keywords were, through multivariate co-occurrence analysis allocated to 20 specific clusters^(^ 14 ^)^. Figure 5 provides a brief title and synopsis of each of the clusters.

**Figure 5:** Short title and brief descriptors of co-occurrence analysis of keywords based on analysis of articles extracted from Scopus

Cluster & [No of keywords]	Brief title	Succinct description
One [120]	Physiological aspects of noncommunicable disease	Highlights the physiological impact of diet on non-communicable diseases such as diabetes and hyperlipidemia.
Two [67]	Child health and socio-economic factors	Explores a wide range of economic and environmental factors that impact the physical and psychological health of children.
Three [57]	Leadership regulation and professionalism	Looks at the role of leaders and regulation on the work environment, care delivery and the structure and quality of services.
Four [49]	Chronic conditions and health maintenance	Details several chronic conditions and the impact for self and family in wellbeing and health maintenance
Five [44]	Healthy eating	Examines a wide range of aspects of healthy eating including food content, portion size and energy intake and expenditure.
Six [42]	Universal health coverage	Looks at a wide range of issues associated with universal health coverage and the role of the nurse in delivering it.
Seven [42]	Collaboration and teamwork	Highlights the importance of collaboration in delivering access to quality services and innovative health services reform.
Eight [40]	Stress and physiological & psychological response	Includes a range of triggers and both normal and abnormal mechanisms to coping with stress.
Nine [39]	Cardiovascular disease	Focuses on conditions relating to cardiovascular disease and the role of diet as a contributory factor.
Ten [34]	End of life care and euthanasia	Explores psychosocial aspects of end of life care, suicide and euthanasia.
Eleven [33]	Childhood and adolescent obesity	Examines the impact of fast food and sugar-sweetened beverages on children and adolescents of all ages.
Twelve [33]	Interpersonal violence	Focus on children and older persons that are subjected to physical or psychological abuse and how this can be identified and addressed.
Thirteen [32]	Health inequalities and under-served populations	Highlights a range of issues that impact of the delivery of services in rural and disadvantaged communities.
Fourteen [31]	Residential and long-term care of the older person	Explores the role of advanced practice nursing in the delivery of comprehensive patient-centered care coordination of the older adult.
Fifteen [30]	Ethics	Focuses on ethics, decision making and informed consent dealing with often new interventions and or contentious and sensitive issues
Sixteen [28]	Early childhood nutrition	Examines breast feeding and healthy nutrition of newborn and young children and its relationship to growth and development.
Seventeen [27]	Mental health of the older adult	Looks at a range of mental health issues and their sequalae withing populations of older adults.
Eighteen [24]	Cultural aspects of breast and cervical cancer	Addresses cultural issues associated with access to physical and psychological treatment for breast and cervical cancer services within Hispanic communities.
Nineteen [21]	Primary and community health advocacy	Focuses on various dimension of advocacy for primary and community health care provision.
Twenty [20]	Life and death dilemmas	Identifies a range of situations where there is increased risk associated with life, death or significant adverse outcomes.

## Discussion

It can be seen from Figure 1 that there has been a significant growth in advocacy work within countries of the Pan American Health Region. The high R-value associated with the growth curve provides a good basis for future projections and can be used as a baseline to identify whether the profession rises to the challenges set out by the Nursing Now Campaign. Exceeding predicted performance based on calculating the numbers of papers projected to be published in the years ahead would indicate that the profession has used the opportunity offered by WHO’s recognition of 2020 as the International Year of the Nurse and Midwife to spring-board efforts to increase their policy voice into the future.

To do this in a purposeful manner would require us to address some of the weaknesses revealed in Figures 2 and 3. By building on existing networks of authors to strengthen connections across the PAHO network would increase diversity of contributions and enhance the potential to conduct multi-center studies. Such an approach would potentially increase study sample sizes and enhance the possibility of generalizability of findings. To do this, existing authors should strategically reach out to less engaged countries and make connections outside their immediate network and subnetwork of co-authors. Authors should feel empowered to use these results to identify potential collaborators. This would further strengthen the existing networks and significantly reduce the likelihood of fragmentation of existing structures. Inevitably, with such a diverse region, language needs to be considered as a potential barrier to accomplishing collaborative work. Indeed, in a study of the social networks of nurse leaders^(^ 16 ^)^ it was identified that factors associated with the establishment of peer to peer connections was related to geographic proximity, language and participation in professional associations. In a subsequent study, the same authors^(^ 17 ^)^ identified that technology can be used to maintain links between participants of such networks. Furthermore, although not ideal, the advancement of technology based translating services may help alleviate or even eliminate some of the historical barriers that authors who speak different languages have faced. Certainly, we can build on existing strengths such as multi-lingual journals like the Revista Latino-Americana de Enfermagem (RLAE). By publishing in such journals, a nexus for collaboration is produced as well as offering a platform that facilitates the identification of authors from English, Spanish and Portuguese speaking countries.

Turning to Figure 4, and more specifically, Figure 5, there is already a wealth of scholarship that focuses on priority topics that will support PAHO in pursuing a wide range of strategic goals. It is therefore essential that when addressing these challenges, the leaders of policy development groups actively seek out and engage nursing expertise.

Figure 5 succinctly illustrates that the policy advocacy efforts of nursing in the PAHO region is well aligned with important health priorities such as non-communicable diseases and related factors such as nutrition, obesity and exercise. Multiple reports at national, regional and global levels have identified the catastrophic impact that such diseases and life-style choices can have on the health, wellbeing and the economy of individuals and nations^(^ 18 ^)^. Nursing in its advocacy efforts in the region have clearly prioritized this topic as seen by its visibility within the scholarship space.

Childhood challenges seen through the lens of the social determinants of health is also well addressed by the profession as is issues associated with addressing the social determinants and the health inequalities faced by underserved and hard to reach communities. At the other end of the spectrum it is encouraging to note that nurse scholarship is also focusing upon some of the most ethically sensitive issues of today. Life-and-death dilemmas, end of life care and euthanasia, along with wider dimensions of ethical decision-making, illustrate the willingness of the profession to address some of the most challenging and sensitive topics faced by society.

With progress on the sustainable development goals and in particular universal health coverage high on the priority agenda for many nations it is important to acknowledge that universal health coverage (UHC) and primary and community health advocacy has emerged as focal issues. Nurses must use the evidence generated to promote their role in the attainment of UHC. However it is only by ensuring that nurses work to their full scope of practice as envisioned by the UN High-Commission on Health Employment and Economic growth that the health and financial benefits will be fully realized^(^ 19 ^-^ 20 ^)^.

Another thorny topic that scholarship in the region is addressing, a far too common global problem, is interpersonal violence. Whether it relates to the abuse of children, the elderly, families or in the work environment nurses are highlighting how it can be identified and addressed.

The final two dominant topics identified through this analysis are central to the mobilization and delivery of change. Specifically, collaboration and teamwork and the cluster associated with leadership, regulation and professionalism. Coupled with the efforts to advocate and influence policy change it is these two areas of attention that will provide the underpinning force to realize policy shifts. These findings align well with the aspirations identified by the chair of the Nursing Now Campaign^(^ 21 ^)^in his interview addressing how the nursing profession can support the pursuit and delivery of UHC.

Obtaining a clear understanding of the current status of nursing scholarship from the published works of nurses in PAHO is important but so is the need to identify what is missing. While it could be argued that some of these observations could well be embedded in the detail of many of the clusters already present, it is perhaps important to call them out. At a time of such rapid change the need for education reform that ensures that the next generation is prepared for the future rather than the past is conspicuous by its absence. Also, the role of technology in facilitating care delivery as well as some of the ethical challenges that the public, profession and policy makers will face seeking to protect confidentiality in what is an ever more connected world must be addressed. This coupled with the increased introduction of artificial intelligence, smart monitoring and even robotics are all worthy of consideration, advocacy and policy dialogue.

Finally, and perhaps not surprisingly, there is still little evidence of the profession’s contribution to the wider sustainable development goals. Climate change is having a profound impact on disease patterns, the frequency and intensity of natural disasters and even the availability and health of fish in the oceans that are needed to support calls for the consumption of healthy diets. The profession has already demonstrated from these results that it has a policy advocacy voice. It is up to this and the next generation of nurses to ensure that we not only focus on clinical issues but also diversify and intensify our contribution to these wider determinants of health^(^ 22 ^)^.

There are limitations to this study. Using a single bibliographic database, Scopus, does mean that the journals that are not curated by the database have been excluded. The impact of this is to underestimate the current level of connectivity of scholars in the region. Having said this, this paper nevertheless does offer an important initial benchmark against which with quantitative (the numbers of papers produced and the robustness and extent of networked connections) as well as qualitative (the themes being addressed) can be measured.

## Conclusion

For the first time, a bibliometric analysis of the scholarship activity of nurse advocacy in the Pan American Health Region has been conducted and demonstrated that there is a wealth of contributions already being made that are well aligned to existing health priorities. The existing output can be accurately described through the application of a polynomial equation and hence sets a trend line that can be projected into the future to assess whether future scholarship maintains or even exceeds the current trajectory. Importantly, there are weaknesses that need to be addressed not least the need to diversify contribution from under-represented member states as well as considering how to apply the existing advocacy strengths to new and wider topics. Only time will tell whether the audacious step by the World Health Organization of declaring 2020 as the International Year of The Nurse and Midwife will have a lasting impetus in accelerating the policy contributions of the profession in the years ahead. For now, we have an important benchmark as well as a focus for celebrating the professions contributions to the art and science of nursing.
